# Blood culture positive sepsis in England, 2017–2018: epidemiological assessment of the commissioning for quality and innovation (CQUIN) sepsis indicator

**DOI:** 10.1186/s12879-025-11539-5

**Published:** 2025-09-26

**Authors:** Ranya Mulchandani, Simon Packer, Joshua Howkins, Carla Robinson, Theresa Lamagni, Alex Bhattacharya, Rosy Reynolds, Andre Charlett, Colin Brown, Russell Hope, Susan Hopkins, Isabel Oliver

**Affiliations:** 1 UK Health Security Agency, London, UK; 2 National Institute for Health and Care Research (NIHR) Health Protection Research Unit (HPRU) in Behavioural Science and Evaluation, University of Bristol, Bristol, UK; 3 University of Bristol, Bristol, UK; 4 NIHR HPRU in Healthcare-Associated Infections & Antimicrobial Resistance, UK Health Security Agency, Imperial College London, London, UK

**Keywords:** Sepsis, Blood culture, Bacteraemia, Surveillance, CQUIN

## Abstract

**Background:**

Sepsis remains a significant clinical and public health concern, necessitating timely identification and targeted management for improved patient outcomes. This study describes the epidemiology of sepsis in emergency department attendees across England by analysing a unique multi-site linked dataset to inform approaches to strengthen surveillance and improve our understanding of clinical outcomes.

**Methods:**

An existent study dataset was utilised comprising a sample of paediatric and adult emergency department admissions screened for community-onset sepsis in the Commissioning for Quality and Innovation (CQUIN) program in the 2017/18 financial year linked to Hospital Episode Statistics and Office for National Statistics death registrations. This dataset was linked to the United Kingdom Health Security Agency’s Second-Generation Surveillance System for microbiological data. Descriptive analyses were conducted to characterise sepsis screen positives and negatives in CQUIN, including demographic characteristics, clinical presentations, microbiological profiles, and clinical outcomes.

**Results:**

Of the 4,027 sepsis-screened emergency admissions included, 2,454 (60.9%) were sepsis screen positive under the CQUIN indicator. Only 11.2% (453/4,027) had a positive blood culture within 2 days of hospital admission. Blood culture positivity rates were 15.2% (373/2,454) and 5.1% (80/1,573) for sepsis screen positive and negative in CQUIN, respectively. Monomicrobial episodes predominated (86.5%), with *Escherichia coli* and *Staphylococcus* species being the most commonly isolated bacteria. The study showed a case fatality rate of 17.1% (420/2,454) for sepsis screen positive in CQUIN but revealed no significant difference in all-cause 30-day mortality between sepsis screen positives in CQUIN with and without positive blood cultures. Sepsis screen positives in CQUIN with a focal site of infection code were more likely to have positive blood cultures, except for respiratory infections.

**Conclusions:**

This study provides novel insights into the epidemiology of sepsis screening in emergency departments across England, highlighting variability in blood culture positivity rates and microbial profiles. The findings underscore the importance of enhanced surveillance strategies, optimised screening protocols, tailored antimicrobial stewardship practices, and quality improvement initiatives to optimise sepsis management and outcomes. Systemic approaches are needed to address knowledge gaps and inform evidence-based interventions for sepsis care.

**Supplementary Information:**

The online version contains supplementary material available at 10.1186/s12879-025-11539-5.

## Introduction

Sepsis is defined as life-threatening organ dysfunction caused by a dysregulated host response to infection [1]. Sepsis is a significant clinical and public health concern due to its high associated mortality, healthcare costs and long term physical, psychological, and cognitive sequelae [2]. In 2022, sepsis was the reported cause of 3,770 deaths in England and Wales and noted on death certification as being an underlying or contributory factor in a further 25,542 deaths [3]. Sepsis has a hospital mortality rate of around one-third (36%) [4–7].

Rapid identification of patients with sepsis is critical for targeted clinical management [8]. It can be clinically screened for using a variety of scoring systems such as National Early Warning Score (NEWS2), the quick Sequential Organ Failure Assessment (qSOFA), Systemic Inflammatory Response Syndrome (SIRS) criteria, among others. However, each system varies in sensitivity, specificity, and clinical utility [9] due to variations in clinical coding, case definitions and use in clinical practice. Additionally, as they do not identify the causative organism, they are unable to guide appropriate choice of antibiotics and patients must first be treated empirically [10]. Therefore, blood culture remains the most important microbiological investigation in the management of sepsis, allowing identification of the responsible organism and direct targeted investigations for underlying source unless this is clinically evident [11]. As such, blood cultures drawn before administration of broad-spectrum antibiotics and within 3 h of a sepsis diagnosis forms a key component of the Surviving Sepsis Campaign care bundle [12, 13].

There is currently no dedicated surveillance system for sepsis in England, limiting our ability to quantify sepsis burden and monitor efforts in reducing its occurrence and impact. Current estimates of sepsis incidence in England are primarily derived from bespoke analysis of routine administrative data, such as Hospital Episode Statistics (HES) using International Classification of Disease (ICD-10) codes to record clinical diagnoses [14]. This system, which contains details on all admissions at National Health Service (NHS) hospitals in England, is primarily designed to provide financial reimbursement for hospitals, and therefore its secondary use for surveillance has limitations, including imprecise clinical coding of infection [2, 15, 16]. Additionally, it is often difficult to translate complex conditions, such as sepsis, into a single ICD-10 code, and the use of these codes have previously been found to have low specificity and sensitivity [16]. While UK Health Security Agency (UKHSA, formerly Public Health England [PHE]) has developed a national surveillance system to automate the reporting of positive blood isolates from diagnostic laboratories in England, the reason for the blood culture collection is not routinely recorded or collated.

In 2015 Commissioning for Quality and Innovation (CQUIN) indicators on sepsis were introduced to incentivise its screening in patients that arrive in emergency departments (EDs) and acute inpatient settings [17]. The CQUIN indicator measured the percentage of adult and child patients arriving in hospital as emergency admissions or on acute in-patient wards who met the National Institute for Health and Care Excellence (NICE) guidance for recognition of sepsis and were screened for sepsis [18], including review by a senior clinical decision maker, though precise criteria for screening varied by local protocol [19]. From 2018, all acute in-patient wards and EDs were recommended to use the NEWS2 system for clinical screening of acutely ill patients; prior to this, local guidance varied widely on the criteria applied for screening patients for sepsis and case definitions used [19]. A version of the CQUIN sepsis indicator ran until 2019. The aim of the indicator was to encourage the timely identification of the causative agent and administration of antibiotic treatment, to improve patient outcomes and reduce sepsis-associated mortality and morbidity.

The data collected during the implementation of the CQUIN sepsis indicator in hospitals provides a novel data source to further our understanding of sepsis epidemiology in England. Additionally, it allows a means to further understand and validate other routine data sources, such as HES, for identifying sepsis. Finally, it provides a rich resource which can be further linked to other datasets, to provide further insights into the causative pathogens in people treated for sepsis in England. This paper aims to combine data from CQUIN and HES with UKHSA’s Second Generation Surveillance System (SGSS) for positive microbiology results, to describe the bacterial and fungal organisms isolated from blood cultures in a sample of patients screened for sepsis in CQUIN and attempt to identify associations between positive bloodstream isolates and clinical outcomes.

## Methods

### Data collection and management

This study builds on previous research conducted within UKHSA, which aimed to assess the feasibility of using existing data for sepsis surveillance in England. A dataset was created combining data from three sources: CQUIN, HES and Office for National Statistics (ONS) [3, 14, 17]. Full details on the dataset curation have been described elsewhere [20]. In summary, the dataset contained a random sample of CQUIN sepsis screened admission records from 30 Hospital Trusts, including NHS number (unique patient identifier), demographic data, and clinical admission data, as well as 30-day all-cause mortality from the ONS. Only individuals screened in, and admitted from, EDs into hospital were included. Each record related to an admission ‘super spell’: a continuous period of inpatient care. Persons could be admitted on multiple occasions over the study period. Trusts’ submissions varied in size and the study used random selection of data submitted by each trust stratified by CQUIN sepsis screen result (positive/negative) with 110 randomly selected in each stratum or, if fewer than 110, all available in that stratum.

**Fig. 1:**
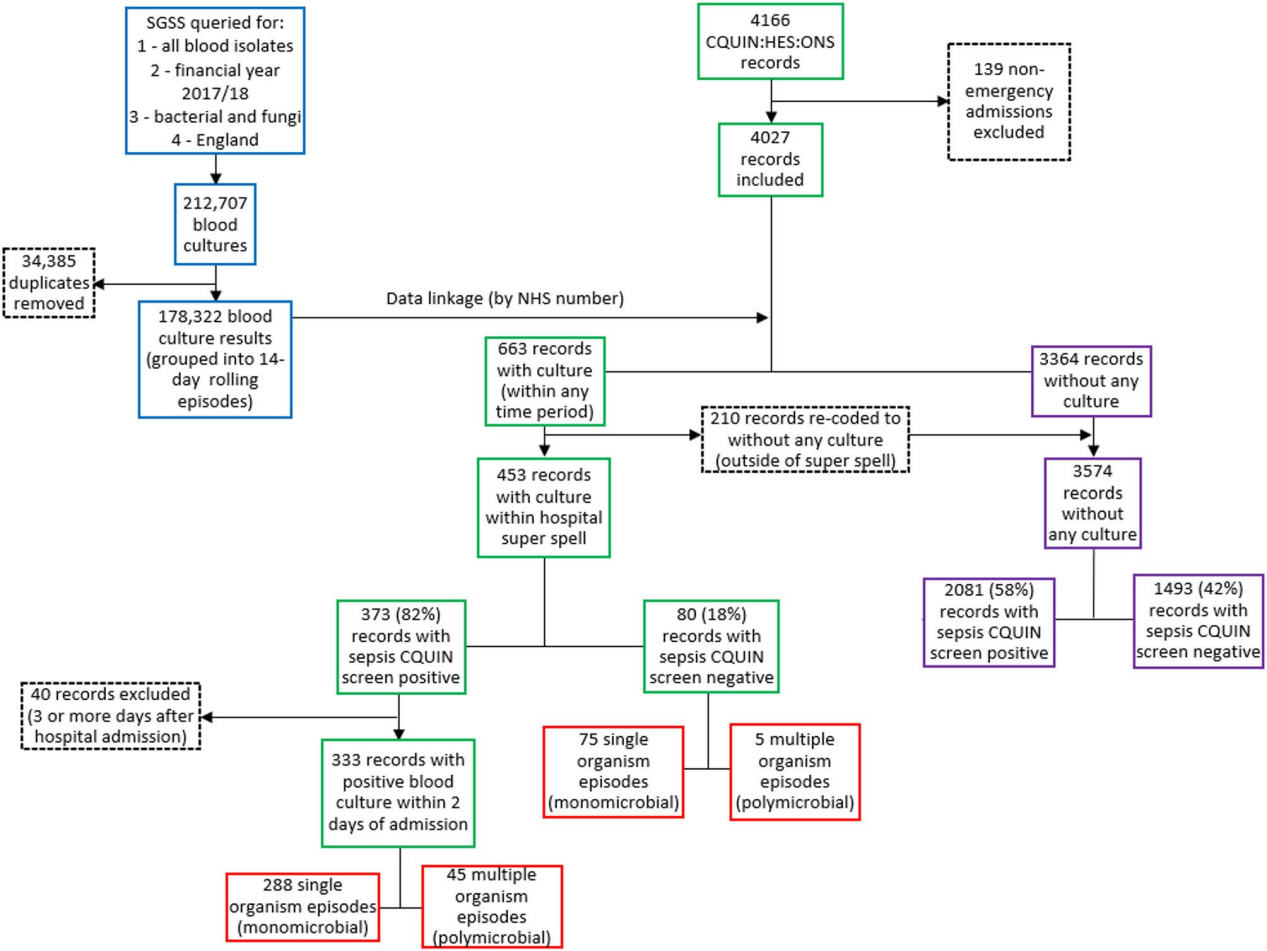
Flow diagram of exclusions and data linkage of study dataset with blood isolate episodes. Figure legend: Study dataset contains data from CQUIN, HES, and ONS; blood isolate episodes from SGSS, CDR feed; linked together to create linked study dataset

In this study, this dataset was further enhanced through deterministic linkage by NHS number with routine laboratory surveillance data to obtain microbiological results, to create the full linked study dataset (Fig. 1). First, any records not coded as emergency admissions (i.e., admission to hospital not coming through the emergency department such as elective surgery and maternity) in hospital admission method in HES were dropped. Then, positive isolates were obtained from the Communicable Disease Record (CDR) data feed of the SGSS held by the UKHSA. The CDR feed was queried for all positive blood isolate records (bacteria and fungi) between 25 March 2017 and 05 April 2018 (to allow for a 5-day lag and lead time on specimen date). Isolate records were grouped into rolling 14-day episodes (i.e., cultures within 14 days were grouped together with the 14 days resetting until there were no more cultures within a subsequent 14-day period) by unique patient identifier (based on NHS number, hospital number, specimen date and date of birth). All positive blood cultures were included in the analysis, without any differentiation based on clinical significance or contamination.

Episodes were retained where the first isolate record fell within 2 days before and after the patient’s first date of their hospital admission ‘super-spell’, defined in HES as a continuous admission period including transfer to receiving hospitals if applicable, as the infection was considered the same episode of hospitalisation and defined as ‘community-onset’. Any records with a first positive blood culture within three or more days after the admission the hospital admission were considered ‘hospital-onset’ and were not included in the descriptive analysis.

Monomicrobial episodes were classified as those with isolates from a single species within the 14-day period. Polymicrobial episodes were classified as those with multiple isolates of different species, from one or more blood cultures, within the 14-day episode.

#### The following definitions were employed

1. Sepsis screen in CQUIN (positive or negative): Persons presenting at EDs with suspected sepsis (based on local protocols) who underwent a sepsis screen as part of the CQUIN sepsis indicator and screened either positive or negative for sepsis.
2. Sepsis in primary HES code (present or absent): Persons included in [1], and who had (present) vs. those who did not have (absent) a primary diagnosis of sepsis in the first Finished Consultant Episode from HES (main condition treated during an episode of care). ICD-10 codes A40.x and A41.x were used in this analysis, as used elsewhere [15, 16, 21]. ICD-10 codes R57.2 and R65.1 were grouped and included as ‘Septic shock/SIRS with organ dysfunction’.
3. Positive blood culture (present or absent): Persons included in [1], and who had (present) vs. those who did not have (absent) a positive blood culture record present within UKHSA’s SGSS CDR feed within 2 days before and after initial date of hospital admission ‘super-spell’.

30-day all-cause mortality was determined as a person included in [1] that had an all-cause death recorded in ONS in the thirty days following the date of their hospital admission.

### Data analysis

The linked dataset was managed, cleaned, and analysed in R version 3.5.0 [22] including use of the ‘Tidyverse’ package [23]. Descriptive analyses were undertaken on presence of corresponding positive blood culture results by demographic, admission and clinical variables (obtained from CQUIN, HES, ONS and/or SGSS), and outcome (obtained from SGSS or ONS). The primary outcome was detection of any bacteria or fungi in blood cultures. Categorical variables were compared using chi-squared test and continuous variables compared by Mann-Whitney test.

## Results

### Description of original study dataset

Of the 4,166 records in the original study dataset, 139 were removed as non-emergency admissions. Of the remaining 4,027 records, 2,454 (60.9%) were sepsis screen positive and 1,573 (39.1%) sepsis screen negative in CQUIN. Of the 2,454 sepsis screen positive in CQUIN, around half (50.5%) were male. The majority (88.0%) were of white ethnicity, while the remaining 6.0% were non-white, with 6.0% of unknown ethnicity. Around a third (34.4%) of sepsis screen positives in CQUIN were over the age of 80; 48.0% were between the ages of 50 and 79, and 7.1% were under the age of 18 (Table 1). The majority (96.1%) had admission method recorded in HES as Accident & Emergency (only 96 were referred through General Practice or other) and the main speciality of patient’s consultant once admitted as general medicine (83.2%). Eighty-four per cent were subsequently discharged from hospital; 420 sepsis screen positives in CQUIN (17.1%) died (all-cause) within 30 days of their hospital admission (Table 1). There were 49 individuals with more than one hospital admission ‘super spell’, accounting for 1.3% (*n* = 101) of all records.

**Table 1:** Characteristics and outcomes of sepsis screen positives in CQUIN emergency admissions by blood culture results

		Positive blood culture		
	Sepsis screen positives in CQUIN (*n* = 2454)	Presence (*n* = 333)	Absence (*n* = 2121)	*p*-value (chi^2^)
Demographic characteristics, *n* (%)				
Sex Male Female	1240 1214	190 (15.3%) 143 (11.8%)	1050 (84.7%) 1071 (88.2%)	0.010 (6.57)
Ethnicity White All other ethnic groups combined^ Unknown	2159 147 148	291 (13.5%) 21 (14.2%) 21 (14.3%)	1868 (86.5%) 127 (85.8%) 126 (85.7%)	0.938 (0.128)
Age (years), median (IQR)	73 (59–83)	75 (64–84)	73 (58–83)	0.008
Age group 0–17 18–49 50–69 70–79 80+	174 260 605 572 843	19 (10.9%) 20 (7.7%) 82 (13.6%) 82 (14.3%) 130 (15.4%)	155 (89.1%) 240 (92.3%) 523 (86.4%) 490 (85.7%) 713 (84.6%)	0.022 (11.4)
Primary HES code*, *n* (%)				
Sepsis Present Absent	665 1789	159 (23.9%) 174 (9.7%)	506 (76.1%) 1615 (90.3%)	< 0.001 (83.2)
Clinical characteristics – site of infection**, *n* (%)				
Respiratory Yes	1220	127 (10.4%)	1093 (89.6%)	< 0.001 (20.7)
Genitourinary Yes	368	98 (26.6%)	270 (73.4%)	< 0.001 (63.0)
Gastrointestinal Yes	78	18 (23.1%)	60 (76.9%)	0.020 (6.33)
Skin and subcutaneous tissue Yes	165	36 (21.8%)	129 (78.2%)	0.001 (10.3)
Other Yes	115	40 (34.8%)	75 (65.2%)	< 0.001 (46.3)
Complications				
Septic shock/SIRS with organ dysfunction*** Yes No	118 2336	26 (22.0%) 307 (13.1%)	92 (78.0%) 2029 (86.9%)	0.006 (7.57)
30-day mortality****, *n* (%)				
Yes	420	65 (15.5%)	355 (84.5%)	
No	2034	268 (13.2%)	1766 (86.8%)	0.21 (1.57)

Legend: *A40.x and A41.x; **HES site of infection codes; ***R57.2 and R65.1; ****all-cause mortality in ONS

### Description of linked study dataset

Of all the 4,027 records (CQUIN screen positive or negative), 453 (11.2%) linked to a positive blood isolate within the 2-days before and after their hospital admission (Fig. 1): 373 (82.3%) were sepsis screen positive and 80 (17.6%) were sepsis screen negative in CQUIN (Fig. 1). Heterogeneity was observed in blood culture positivity rates by CQUIN and HES classifications (Table 2). There was no evidence for a statistically significant difference in 30-day all-cause mortality between those with and without presence of positive blood culture (15.5% vs. 13.2%, *p* = 0.21) (Table 1).

### Blood culture positivity by sepsis classification

Sepsis screen positives in CQUIN had higher proportions of positive blood culture as compared to sepsis screen negatives in CQUIN (15.2% vs. 5.1%, *p* < 0.001). In addition, heterogeneity was seen within these groups when stratified by presence or absence of sepsis in a primary HES code (Table 2).

**Table 2:** Sepsis screen positives in CQUIN with at least one positive blood culture during admission

		Positive blood culture*		
		**Presence (%)**	**Absence (%)**	**Total**
Sepsis screen in CQUIN	Sepsis in primary HES code			
Positive	All	373 (15.2)	2081 (84.8)	2454
	Present	168 (25.3)	497 (74.7)	665
	Absent	205 (11.5)	1584 (88.5)	1789
Negative	All	80 (5.1)	1493 (94.9)	1573
	Present	18 (14.1)	110 (85.9)	128
	Absent	62 (4.3)	1383 (95.7)	1445
Total	All	453 (11.2)	3574 (88.8)	4027

Legend: At least one culture result available in SGSS. Split by presence/absence of sepsis in a primary HES code (*n*=4027)

*as reported in UKHSA’s SGSS

### Microbiological characteristics by sepsis classification

Of the 333 sepsis screen positives in CQUIN with a positive blood culture within two days of hospital admission, 288 (86.5%) were monomicrobial episodes and 45 (13.5%) were polymicrobial. Of the 288 sepsis screen positives in CQUIN with monomicrobial infection, all had bacterial infections (Supplementary Table 1). Of these, 140 (48.6%) also had sepsis recorded in a primary HES code. The top five species identified were *Escherichia coli* (*E. coli;n* = 89; 31%), coagulase-negative *Staphylococcus* (*CoNS*; *n* = 53; 18%), *Streptococcus pneumoniae* (*S. pneumoniae;n* = 22; 8%), *Staphylococcus aureus* (*S*. *aureus;n* = 18; 6%) and *Klebsiella pneumoniae* (*K*. *pneumoniae;n* = 16; 6%) (Table 3). As the numbers of records were too small to determine pathogen-specific 30-day all-cause mortality, bacteria were grouped into Gram positive or negative. For the 288 records with monomicrobial episodes, there was no evidence of a difference in 30-day mortality amongst those infected with Gram positive vs. Gram negative bacteria (16% vs. 20% respectively, *p* = 0.57).

**Table 3:** Sepsis screen positives in CQUIN with positive blood culture for monomicrobial episodes

Organism species name	Sepsis in primary HES code		
	Present	Absent	Total
*Escherichia coli*	52 (37.1%)	37 (25.0%)	89 (30.9%)
*Staphylococcus* (coagulase negative)	18 (12.9%)	35 (23.6%)	53 (18.4%)
*Streptococcus pneumoniae*	12 (8.6%)	10 (6.8%)	22 (7.6%)
*Staphylococcus aureus*	7 (5.0%)	11 (7.4%)	18 (6.2%)
*Klebsiella pneumoniae*	10 (7.1%)	6 (4.1%)	16 (5.6%)
*Pseudomonas aeruginosa*	8 (5.7%)	5 (3.4%)	13 (4.5%)
*Streptococcus* group A	2 (1.4%)	6 (4.1%)	8 (2.8%)
*Enterococcus faecalis*	3 (2.1%)	4 (2.7%)	7 (2.4%)
*Streptococcus* alpha and non-haemolytic	5 (3.6%)	2 (1.4%)	7 (2.4%)
*Micrococcus luteus (sarcina)*	2 (1.4%)	3 (2.0%)	5 (1.7%)

Legend: Results split by presence/absence of sepsis in primary HES code and species (Top 10, *n* = 288)

Of the 148 (51.4%) blood culture positive (monomicrobial) sepsis screen positives in CQUIN that did not have sepsis in a primary HES code, the most common HES primary infection ICD-10 codes were for “Lobar pneumonia, unspecified organism” (*n* = 12; 8.1%), “Pneumonia, unspecified organism” (*n* = 9; 6.1%), Pneumonia due to S. *pneumoniae* (*n* = 7; 4.7%), “Pneumonitis due to inhalation of food and vomit” (*n* = 7; 4.7%), “Cellulitis and acute lymphangitis of other parts of limb” (*n* = 7; 4.7%) and “Urinary tract infection, site not specified” (*n* = 7; 4.7%) (Supplementary Table 2).

Of the 45 sepsis screen positives in CQUIN with positive blood culture results from polymicrobial episodes, all cultures were bacterial —not fungal—and made up a total of 98 species isolates (Table 4). Of these, eight individuals had three different organisms within one episode (an episode being 14-day rolling window of blood culture), 36 individuals had two different organisms within one episode and one record had one organism in their first episode and one (different) organism in their second episode (within the same super-spell). Of these 98 isolates, the most observed species were *E. coli* (*n* = 16; 16.3%), *CoNS* (*n* = 15; 15.3%), coliforms excluding *E. Coli* (*n* = 8; 8.2%), *Enterococcus faecalis (E. faecalis;n* = 4; 4.1%), *Proteus mirabilis (P. mirabilis;n* = 4; 4.1%) and *S. aureus* (*n* = 4; 4.1%). None of the sepsis screen negatives in CQUIN had a polymicrobial episode.

**Table 4:** Sepsis screen positives in CQUIN with positive blood culture for polymicrobial episodes (Top 10)

Organism species name	Total
*E. coli*	16 (16.3%)
*Staphylococcus (coagulase negative*,* CoNS)*	15 (15.3%)
Coliforms excluding *E. Coli*	8 (8.2%)
*E. faecalis*	4 (4.1%)
*P. mirabilis*	4 (4.1%)
*S. aureus*	4 (4.1%)
*Enterococcus faecium*	3 (3.1%)
*K. pneumoniae*	3 (3.1%)
*Streptococcus alpha and non-haemolytic*	3 (3.1%)
*Bacteroides fragilis*	2 (2.0%)

Legend: (*n* = 45 records with 98 isolates)

There was evidence to suggest that sepsis screen positives in CQUIN that had a site of infection code in HES were more likely to have presence of a positive blood culture (Table 1), except for respiratory infection where they were less likely to have a presence of positive blood culture (*p* < 0.001) (Table 1). Being male or over 50 years of age were also associated with higher proportions of positive blood cultures, as was having sepsis as HES primary code (Table 1). When looking specifically at sepsis screen positives in CQUIN recorded with respiratory tract as site of infection (*n* = 115), 43 (37.4%) also had sepsis in a primary HES code. The most common species were *CoNS* (*n* = 31; 27.0%), *E. coli* (*n* = 21; 18.3%) and *S. pneumoniae* (*n* = 17; 14.8%). For records coded with a respiratory site of infection, there was no evidence for a difference in 30-day mortality amongst those infected with Gram positive or negative bacteria, respectively (29% v 18%, *p* = 0.274).

## Discussion

This study provides insights into the epidemiology of sepsis in emergency departments across England. In this study, we found that of patient admission records with sepsis screens in CQUIN in our linked study dataset, only 11.2% had a positive blood culture within 2 days of hospital admission, though SGSS only includes positive culture results. Positivity proportions varied by both CQUIN and HES sepsis classifications; for instance, sepsis screen positives in CQUIN had higher positivity proportion than sepsis screen negatives in CQUIN. The positivity proportion is lower than previous studies, such as by Bernard et al. who observed 32%, Phau et al. with 42%, and Panday et al. with ~ 43% [24–26]. However, these studies focused on cases defined as severe sepsis or using pre-hospital diagnostic criteria rather than the screening of all potential cases of sepsis within a hospital setting, where sensitivity for sepsis may be prioritised clinically [24–26]. We found that sepsis CQUIN screen positives with a noted site of infection code in HES were more likely to have a positive blood culture, except for respiratory disease who were less likely to have a positive blood culture. This may be due to greater clinical certainty of the differential diagnosis when a clear source of infection is observed, except in respiratory infections who may be less likely to become bacteraemic [27]. These findings add to recent evidence of phenotypic variation in illness severity in sepsis when stratified by infection site [28].

Most of the blood culture positive episodes identified were monomicrobial, with the most common organisms *E. coli* and *CoNS*, though note that many *Staphylococcus isolates* are likely to be contaminants [29]. There was no evidence for a statistically significant difference in 30-day mortality between those with and without presence of positive blood culture. This is in line with Sigakis et al., who found positive culture was not independently associated with mortality (OR 1.01, 95% CI 0.81–1.26) and Yang et al., who found positive blood culture was not associated with either in-hospital mortality or 60-day mortality but was associated with increased length of stay in hospital and in Intensive Care Units (*p* = 0.007 and *p* = 0.016, respectively) [30, 31]. Conversely, this differs from findings by Panday et al. who found a significant difference in 28-day mortality between culture-positive and culture-negative patient groups (RR 1.43, 95% CI 1.11–1.83) [26]. These differences may underscore the importance of independent factors on risk of mortality in sepsis, and the need for further research in this area.

This paper uses a unique multi-site dataset obtained from the CQUIN sepsis indicator, further enhanced by linking together multiple data sources from HES, ONS, and SGSS, allowing an overview of emergency admissions screened for sepsis in CQUIN in England in 2017–2018. This allows for a novel exploration of both the CQUIN sepsis indicator and validation of HES for obtaining information on sepsis, together with detailed description on individual characteristics and main causative bacteria.

There are several limitations to this study which should be considered. Importantly, we did not exclude any positive culture results which may not have been clinically significant, such as those that may be due to contamination. As such, we may have over-estimated the true rate of positive blood cultures that caused the sepsis episode and impacted the sensitivity of our analysis to observe clinically significant relationships [32]. As there was no single definition of sepsis being stipulated in CQUIN, there may have been some heterogeneity in those eligible for screening as part of CQUIN across trusts, which was not possible to assess in this study: as such, we describe individuals throughout the paper as “sepsis screen positive/negative in CQUIN” to emphasise this. UKHSA’s laboratory surveillance system (SGSS) only contains positive blood cultures: as such, for those with an absence of positive blood culture result, it is not possible to ascertain whether this is due to a negative result, perhaps due to preceding antibiotic administration or other factor [33], or to the absence of any culture being taken. Other potential causes of sepsis such as viruses and parasites, as well as non-blood cultures, were not within the scope of this analysis. Therefore, we may have underestimated the number of individuals with a positive culture. Sepsis also frequently occurs in the absence of positive culture results, likely reflecting limitations in current microbiological detection methods and the complex host–pathogen interactions that may not yield identifiable organisms. The 49 persons with multiple admissions (total 101 records) may be a small source of bias due to a higher risk of presenting with a previously identified organism. By considering all-cause 30-day mortality we may have included deaths due to causes other than sepsis. It was not possible to investigate pathogen-specific 30-day mortality due to low numbers: as such, we categorised organisms into two groups, Gram positive and negative, and compared mortality rates between them. However, the utility of this approach is limited due to the heterogeneity of clinical presentations of organisms within these groups (more details on risk factors associated with mortality can be found in Robinson et al. [20]). Finally, administrative systems such as HES and ONS have their own limitations, such as time of coding relative to screening and diagnosis, and low specificity of coding, which impedes ability to compare these datasets [15].

## Conclusion

Our paper highlights the importance of a multi-faceted approach to the identification of sepsis: although positive blood-cultures remain the clinical “gold standard” in the identification of bacteraemia, other additional markers of sepsis need to be considered as part of hospital admission screening protocols, for prognosis and surveillance. The finding of only a low proportion of admissions having a positive blood culture within 2 days of hospital admission is particularly significant here, reinforcing that other markers must play a role in guiding screening, management, and surveillance.

There is significant local variation in the application of sepsis screening tools: the development of a clinically optimised sepsis-identification pathway, validated across a range of geographic locales and demographic cohorts, is a logical next step towards improving the early identification of sepsis in England, and ultimately reducing 30-day mortality and the societal costs associated with sepsis.

## Supplementary Information

Supplementary Material 1.

## Data Availability

The dataset analysed during the current study is not publicly available due to inclusion of patient-level pseudonymised data. Data may be available from the corresponding author on reasonable request.

## References

[1] Singer M, Deutschman CS, Seymour CW, Shankar-Hari M, Annane D, Bauer M, et al. The third international consensus definitions for sepsis and septic shock (Sepsis-3). JAMA. 2016;315(8):801–10. 26903338 10.1001/jama.2016.0287 PMC4968574

[2] Cohen J, Vincent J-L, Adhikari NK, Machado FR, Angus DC, Calandra T, et al. Sepsis: a roadmap for future research. Lancet Infect Dis. 2015;15(5):581–614. 25932591 10.1016/S1473-3099(15)70112-X

[3] Office for National Statistics. Deaths from sepsis in the UK 2001 to 2022. 2023. Available from: https://www.ons.gov.uk/peoplepopulationandcommunity/birthsdeathsandmarriages/deaths/adhocs/1468deathsinvolvingsepsisenglandandwales2001to2022. Accessed 16/08/2024.

[4] NHS England. Cross-system sepsis action plan 2017. Available from: https://www.england.nhs.uk/publication/cross-system-sepsis-action-plan-2017/. Accessed 16/08/2024.

[5] Daniels R. Surviving the first hours in sepsis: getting the basics right (an intensivist’s perspective). J Antimicrob Chemother. 2011;66(suppl2):ii11–23. 21398303 10.1093/jac/dkq515

[6] Vincent J-L, Sakr Y, Sprung CL, Ranieri VM, Reinhart K, Gerlach H, et al. Sepsis in European intensive care units: results of the SOAP study. Crit Care Med. 2006;34(2):344–53. 16424713 10.1097/01.ccm.0000194725.48928.3a

[7] Levy MM, Dellinger RP, Townsend SR, Linde-Zwirble WT, Marshall JC, Bion J, et al. The surviving sepsis campaign: results of an international guideline-based performance improvement program targeting severe sepsis. Intensive Care Med. 2010;36:222–31. 20069275 10.1007/s00134-009-1738-3 PMC2826633

[8] Rudd KE, Kissoon N, Limmathurotsakul D, Bory S, Mutahunga B, Seymour CW, et al. The global burden of sepsis: barriers and potential solutions. Crit Care. 2018;22:1–11. 30243300 10.1186/s13054-018-2157-z PMC6151187

[9] Yu SC, Shivakumar N, Betthauser K, Gupta A, Lai AM, Kollef MH, et al. Comparison of early warning scores for sepsis early identification and prediction in the general ward setting. JAMIA Open. 2021;4(3):ooab062. 34820600 10.1093/jamiaopen/ooab062 PMC8607822

[10] Frankling C, Yeung J, Dark P, Gao F. I spy with my little eye something beginning with S: spotting sepsis. Oxford University Press; 2016. p. 279–81. 10.1093/bja/aew254 27543521

[11] Patel M. Utility of blood culture in sepsis diagnostics. J Acad Clin Microbiologists. 2016;18(2):74–9.

[12] Levy MM, Evans LE, Rhodes A. The surviving sepsis campaign bundle: 2018 update. Intensive Care Med. 2018;44:925–8. 29675566 10.1007/s00134-018-5085-0

[13] Gilbert JA. Sepsis care bundles: a work in progress. Lancet Respiratory Med. 2018;6(11):821–3. 10.1016/S2213-2600(18)30362-X 30150012

[14] Hospital Episode Statistics (HES). Available from: https://digital.nhs.uk/data-and-information/data-tools-and-services/data-services/hospital-episode-statistics. Cited 16/05/2024.

[15] Chin Y, Scattergood N, Thornber M, Thomas S. Accurate coding in sepsis: clinical significance and financial implications. J Hosp Infect. 2016;94(1):99–102. 27318878 10.1016/j.jhin.2016.05.013

[16] Jolley RJ, Sawka KJ, Yergens DW, Quan H, Jetté N, Doig CJ. Validity of administrative data in recording sepsis: a systematic review. Crit Care. 2015;19:1–12. 25887596 10.1186/s13054-015-0847-3 PMC4403835

[17] NHS England. Commissioning for Quality and Innovation. Available from: https://www.england.nhs.uk/nhs-standard-contract/cquin/. Accessed 16/08/2024.

[18] National Institute for Health and Care Excellence. NICE guideline [NG51]. Sepsis: recognition, diagnosis and early management. 2016.

[19] NHS England. CQUIN Indicator Specification Information on CQUIN 2017/18–2018/19. Available from: https://www.england.nhs.uk/wp-content/uploads/2017/07/cquin-indicator-specification-information-january-2019.pdf. Accessed 16/08/2024.

[20] Robinson C, Packer S, Howkins J, Mulchandani R, Lamagni T, Brown C et al. October. Predictors of mortality in emergency admissions screened for sepsis as part of the commissioning for quality and innovation (CQUIN) sepsis indicator: a secondary analysis of a National linked dataset. PREPRINT (Version 1) available at research square. 16 Oct 2024.

[21] Inada-Kim M, Page B, Maqsood I, Vincent C. Defining and measuring suspicion of sepsis: an analysis of routine data. BMJ Open. 2017;7(6):e014885. 28601825 10.1136/bmjopen-2016-014885 PMC5734411

[22] R Core Team. R: A Language and environment for statistical computing. Vienna, Austria: R Foundation for Statistical Computing; 2021.

[23] Wickham H, Averick M, Bryan J, Chang W, McGowan LDA, François R, et al. Welcome to the tidyverse. J Open Source Softw. 2019;4(43):1686.

[24] Bernard GR, Ely EW, Wright TJ, Fraiz J, Stasek JE Jr, Russell JA, et al. Safety and dose relationship of Recombinant human activated protein C for coagulopathy in severe sepsis. Crit Care Med. 2001;29(11):2051–9. 11700394 10.1097/00003246-200111000-00003

[25] Phua J, Ngerng WJ, See KC, Tay CK, Kiong T, Lim HF, et al. Characteristics and outcomes of culture-negative versus culture-positive severe sepsis. Crit Care. 2013;17:1–12. 10.1186/cc12896 PMC4057416 24028771

[26] Nannan Panday RS, Lammers EM, Alam N, Nanayakkara PW. An overview of positive cultures and clinical outcomes in septic patients: a sub-analysis of the prehospital antibiotics against sepsis (PHANTASi) trial. Crit Care. 2019;23:1–9. 31113475 10.1186/s13054-019-2431-8 PMC6530106

[27] Nejtek T, Müller M, Moravec M, Průcha M, Zazula R. Bacteremia in patients with sepsis in the ICU: does it make a difference?. Microorganisms. 2023;11(9):2357 . 10.3390/microorganisms11092357 PMC10534394 37764201

[28] Schertz AR, Eisner AE, Smith SA, Lenoir KM, Thomas KW. Clinical phenotypes of sepsis in a cohort of hospitalized patients according to infection site. Crit Care Explor. 2023;5(8):e0955. 37614801 10.1097/CCE.0000000000000955 PMC10443761

[29] Thylefors JD, Harbarth S, Pittet D. Increasing bacteremia due to coagulase-negative staphylococci: fiction or reality? Infect Control Hosp Epidemiol. 1998;19(8):581–9. 9758060 10.1086/647878

[30] Sigakis MJ, Jewell E, Maile MD, Cinti SK, Bateman BT, Engoren M. Culture-negative and culture-positive sepsis: a comparison of characteristics and outcomes. Anesth Analgesia. 2019;129(5):1300–9. 10.1213/ANE.0000000000004072 PMC7577261 30829670

[31] Yang SC, Liao KM, Chen CW, Lin WC. Positive blood culture is not associated with increased mortality in patients with sepsis-induced acute respiratory distress syndrome. Respirology. 2013;18(8):1210–6. 23692513 10.1111/resp.12121

[32] MacGowan A, Grier S, Stoddart M, Reynolds R, Rogers C, Pike K, et al. Impact of rapid microbial identification on clinical outcomes in bloodstream infection: the RAPIDO randomized trial. Clin Microbiol Infect. 2020;26(10):1347–54. 32220636 10.1016/j.cmi.2020.01.030

[33] Cheng MP, Stenstrom R, Paquette K, Stabler SN, Akhter M, Davidson AC, et al. Blood culture results before and after antimicrobial administration in patients with severe manifestations of sepsis: a diagnostic study. Ann Intern Med. 2019;171(8):547–54. 31525774 10.7326/M19-1696

