# Supplementary material for: Blood culture positive sepsis in England, 2017–2018: epidemiological assessment of the commissioning for quality and innovation (CQUIN) sepsis indicator
Source: BMC Infect Dis. 2025 Sep 26;25:1137. doi: 10.1186/s12879-025-11539-5 (PMC12465823; doi:10.1186/s12879-025-11539-5)
Supplement: Supplementary file 1 — Supplementary Material 1. [file 12879_2025_11539_MOESM1_ESM.docx]

**Supplementary Material**

*Table 1: 30-day all-cause mortality by organism for monomicrobial episodes. Note* *from ONS

|  | 30-day all-cause mortality* | |  |
| --- | --- | --- | --- |
| Organism species name | No | Yes | Total |
| *Escherichia coli* | 74 (83%) | 15 (17%) | 89 (100%) |
| *Staphylococcus (*Coagulase Negative) | 45 (85%) | 8 (15%) | 53 (100%) |
| *Streptococcus pneumoniae* | 20 (91%) | 2 (9%) | 22 (100%) |
| *Staphylococcus aureus* | 15 (83%) | 3 (17%) | 18 (100%) |
| *Klebsiella pneumoniae* | 15 (94%) | 1 (6%) | 16 (100%) |
| *Pseudomonas aeruginosa* | 8 (62%) | 5 (38%) | 13 (100%) |
| *Streptococcus* Group A | 7 (88%) | 1 (12%) | 8 (100%) |
| *Enterococcus faecalis* | 6 (86%) | 1 (14%) | 7 (100%) |
| *Streptococcus* Alpha And Non-Haemolytic | 7 (100%) | 0 (0%) | 7 (100%) |
| *Micrococcus luteus (sarcina)* | 4 (80%) | 1 (20%) | 5 (100%) |
| *Enterobacter cloacae* | 3 (75%) | 1 (25%) | 4 (100%) |
| *Streptococcus* Group B | 3 (75%) | 1 (25%) | 4 (100%) |
| *Streptococcus* Group C | 3 (75%) | 1 (25%) | 4 (100%) |
| *Streptococcus* Group G | 2 (50%) | 2 (50%) | 4 (100%) |
| *Neisseria meningitidis* | 3 (100%) | 0 (0%) | 3 (100%) |
| *Proteus mirabilis* | 1 (33%) | 2 (67%) | 3 (100%) |
| *Citrobacter diversus (C. koseri)* | 1 (50%) | 1 (50%) | 2 (100%) |
| *Micrococcus* Spp. | 1 (50%) | 1 (50%) | 2 (100%) |
| *Propionibacterium freudenreichii* | 2 (100%) | 0 (0%) | 2 (100%) |
| *Acinetobacter haemolyticus* | 1 (100%) | 0 (0%) | 1 (100%) |
| *Bacillus Spp.* | 1 (100%) | 0 (0%) | 1 (100%) |
| *Bacteroides fragilis* | 0 (0%) | 1 (100%) | 1 (100%) |
| *Bacteroides Spp.* | 1 (100%) | 0 (0%) | 1 (100%) |
| *Campylobacter jejuni* | 1 (100%) | 0 (0%) | 1 (100%) |
| *Coliform* | 0 (0%) | 1 (100%) | 1 (100%) |
| *Corynebacterium Spp.* | 0 (0%) | 1 (100%) | 1 (100%) |
| *Corynebacterium Striatum* | 1 (100%) | 0 (0%) | 1 (100%) |
| *Diphtheroids* | 1 (100%) | 0 (0%) | 1 (100%) |
| *Enterobacter asburiae* | 1 (100%) | 0 (0%) | 1 (100%) |
| *Enterobacter cloacae complex* | 1 (100%) | 0 (0%) | 1 (100%) |
| *Fusobacterium nucleatum* | 1 (100%) | 0 (0%) | 1 (100%) |
| *Haemophilus influenzae* | 0 (0%) | 1 (100%) | 1 (100%) |
| *Morganella morganii* | 1 (100%) | 0 (0%) | 1 (100%) |
| *Propionibacterium Spp.* | 1 (100%) | 0 (0%) | 1 (100%) |
| *Proteus Spp.* | 1 (100%) | 0 (0%) | 1 (100%) |
| *Pseudomonas stutzeri* | 1 (100%) | 0 (0%) | 1 (100%) |
| *Staphylococcus Other Named* | 0 (0%) | 1 (100%) | 1 (100%) |
| *Streptococcus alactolyticus* | 1 (100%) | 0 (0%) | 1 (100%) |
| *Streptococcus infantarius subsp coli* | 0 (0%) | 1 (100%) | 1 (100%) |
| *Streptococcus Other Named* | 1 (100%) | 0 (0%) | 1 (100%) |
| *Streptococcus thoraltensis* | 1 (100%) | 0 (0%) | 1 (100%) |
| Total | 236 (82%) | 52 (18%) | 288 (100%) |

*Table 2: HES ICD-10 Groupings / Codings. (Adapted from Inada-Kim M, et al. 2017 [ref in main text]).*

| ***ICD-10 Groupings*** | ***ICD-10 Codes*** |
| --- | --- |
| ***Sepsis in primary HES code*** | ***A40*** *Streptococcal sepsis (incl. A40.0, A40.1, A40.2, A40.3, A40.8, A40.9)*  ***A41*** *Other Sepsis (incl. A41.0, A41.1, A41.2, A41.3, A41.4, A41.5, A41.8, A41.9)* |
| ***Site of infection codes (derived from across all twenty ICD-10 diagnostic codes)*** | |
| ***Respiratory*** | ***Diseases of the respiratory system***  ***J01*** *Acute sinusitis (incl. J01.0, J01.1, J01.2, J01.3, J01.4, J01.8, J01.9)*  ***J02*** *Acute pharyngitis (incl. J02.0, J02.9)*  ***J03*** *Acute tonsillitis (incl. J03.0, J03.9)*  ***J05.1*** *Acute epiglottitis*  ***J06.9*** *Acute upper respiratory infection, unspecified*  ***J13*** *Pneumonia due to Streptococcus pneumoniae,*  ***J14*** *Pneumonia due to Haemophilus influenza,*  ***J15*** *Bacterial pneumonia, not elsewhere classified (J15.0, J15.1, J15.2, J15.3, J15.4, J15.5, J15.6, J15.7, J15.8, J15.9)*  ***J16*** *Pneumonia due to other infectious organisms, not elsewhere classified (incl. J16.0, J16.8)]*  ***J17.0*** *Pneumonia in bacterial diseases classified elsewhere (incl. J17.0, J17.8)*  ***J18*** *Pneumonia, organism unspecified (including J18.0, J18.1, J18.2, J18.8 and J18.9)*  ***J20*** *Acute bronchitis (incl. J20.0, J20.1, J20.2, J20.8, J20.9)*  ***J22*** *Unspecified acute lower respiratory infection*  ***J36*** *Peritonsillar abscess*  ***J39*** *Other diseases of upper respiratory tract (incl. J39.0, J39.1)*  ***J44.0*** *Chronic obstructive pulmonary disease with acute lower respiratory infection*  ***J69*** *Pneumonitis due to solids and liquids (incl. J69.0, J69.8)*  ***J84.9*** *Interstitial pulmonary disease unspecified (interstitial pneumonia NOS)*  ***J85*** *Abscess of lung and mediastinum (incl. J85.1, J85.2, J85.3)*  ***J86*** *Pyothorax (incl. J86.0, J86.9)*  ***J95.0*** *Sepsis of tracheostomy stoma*  ***J98.5*** *Diseases of mediastinum, not elsewhere classified- Mediastinitis* |
| ***Genitourinary*** | ***Diseases of genitourinary system***  ***N10*** *Acute tubulo-interstitial nephritis*  ***N11*** *Chronic tubulo-interstitial nephritis (incl. N11.0, N11.1, N11.8, N11.9)*  ***N12*** *Tubulo-intestitial nephritis, not specified as acute or chronic*  ***N13.6*** *Pyonephrosis*  ***N15.1*** *Renal and perinephric abscess*  ***N15.9*** *Renal tubulo-interstitial disease, unspecified*  ***N30*** *Cystitis, unspecified (including N30.0, N30.8, N30.9)*  ***N34.0*** *Urethral abscess*  ***N39.0*** *Urinary tract infection, site not specified*  ***N41.0*** *Acute prostatitis*  ***N43.1*** *Infected hydrocele*  ***N45*** *Orchitis and epididymitis (incl. N45.0, N45.9)*  ***N48.2*** *Other disorders of penis (incl. N48.1, N48.2)*  ***N49.9*** *Inflammatory disorder of unspecified male genital organ*  ***N61*** *Inflammatory disorders of breast*  ***N70*** *Salpingitis and oophoritis (incl N70.0, N70.9)*  ***N71*** *Inflammatory disease of uterus, except cervix (incl. N71.0, N71.9)*  ***N73*** *Other female pelvic inflammatory diseases (incl. N73.0, N73.1, N73.2, N73.4, N73.9)*  ***N75.1*** *Abscess of Bartholin gland*  ***N76*** *Other inflammation of vagina and vulva (incl. N76.0, N76.1, N76.3, N76.4, N76.8)* |
| ***Gastrointestinal*** | ***Diseases of the digestives system (dental disorders omitted)***  ***K22.3*** *Perforation of oesophagus*  ***K35*** *Acute appendicitis (incl. K35.2, K35.3, K35.8)*  ***K37*** *Unspecified appendicitis*  ***K57*** *Diverticular disease of intestine (incl. K57.0, K57.2, K57.4, K57.8)*  ***K61*** *Abscess of anal and rectal regions (incl. K61.0, K61.1, K61.2, K61.3, 61.4)*  ***K63.0*** *Abscess of intestine*  ***K63.1*** *Perforation of intestine (nontraumatic)*  ***K65.0*** *Acute peritonitis (incl. K65.0, K65.8, K65.9)*  ***K67*** *Disorders of peritoneum in infectious diseases classified elsewhere (all subcategories)*  ***K75.0*** *Abscess of liver*  ***K80.0*** *Calculus of gallbladder with acute cholecystitis/cholangitis (incl.K80.0, K80.1, K80.3, K80.4)*  ***K81*** *Cholecystitis (incl. K81.0, K81.1, K81.8, K81.9)*  ***K82.2*** *Perforation of gallbladder*  ***K83.0*** *Cholangitis*  ***K83.2*** *Perforation of bile duct* |
| ***Skin and subcutaneous tissue*** | ***Diseases of skin and subcutaneous tissue***  ***L00*** *Staphylococcal scalded skin syndrome*  ***L01*** *Impetigo (L01.0, L01.1)*  ***L02*** *Cutaneous abscess, furuncle and carbuncle (incl. L02.0, L02.1, L02.2, L02.3, L02.4, L02.8, L02.9)*  ***L03*** *Cellulitis (including L03.0, L03.1, L03.2, L03.3, L03.8 and L03.9)*  ***L05.0*** *Pilonidal cyst with abscess*  ***L08*** *Other local infections of skin and subcutaneous tissue (incl. L08.0, L08.8, L08.9)*  ***L30.3*** *Infective dermatitis*  ***L53.3*** *Erythema marginatum*  ***L98.0*** *Pyogenic granuloma* |
| ***Other*** | ***Diseases of the nervous system***  ***G00*** *Bacterial meningitis, not elsewhere classified (incl. G00.0, G00.1, G00.2, G00.3, G00.8, G00.9)*  ***G01*** *Meningitis in bacterial diseases classified elsewhere*  ***G04.2*** *Bacterial meningoencephalitis and meningomyelitis, not elsewhere classified*  ***G06*** *Intracranial and intraspinal abscess and granuloma (incl. G06.0, G06.1, G06.2)*  ***Diseases of the circulatory system***  ***I00*** *Rheumatic fever without mention of heart involvement*  ***I01*** *Rheumatic fever with heart involvement (incl. I01.0, I01.1, I01.2, I01.8, I01.9)*  ***I02*** *Rheumatic chorea (incl. I02.0, I02.9)*  ***I33*** *Acute and subacute endocarditis (incl. I33.0, I33.9)*  ***I38*** *Endocarditis, valve unspecified*  ***Diseases of the ear and mastoid process***  ***H60*** *Otitis externa (incl. H60.0, H60.1, H60.2, H60.3)*  ***H66*** *Suppurative and unspecified otitis media (incl. H66.0, H66.4, H66.9)*  ***H67.0*** *Otitis media in bacterial diseases classified elsewhere*  ***H68.0*** *Eustachian salpingitis*  ***H70*** *Mastoiditis and related conditions (incl. H70.0, H70.9)*  ***H73.0*** *Acute myringitis*  ***Diseases of the musculoskeletal system and connective tissue***  ***M00*** *Pyogenic arthritis (incl. M00.0, M00.1, M00.2, M00.8, M00.9)*  ***M01*** *Direct infections of joint in infectious and parasitic diseases classified elsewhere (incl. M01.0, M01.1, M01.2, M01.3)*  ***M46.2*** *Osteomyelitis of vertebra*  ***M46.4*** *Discitis, unspecified*  ***M65*** *Synovitis and tenosynovitis (incl. M65.0, M65.1)*  ***M71.0*** *Abscess of bursa*  ***M72.6*** *Necrotizing fasciitis*  ***M86*** *Osteomyelitis*  ***Pregnancy, Childbirth and the puerperium and certain conditions originating in the perinatal period***  ***O08.0*** *Genital tract and pelvic infection following abortion and ectopic and molar pregnancy*  ***O23*** *Infections of genitourinary tract in pregnancy (incl. O23.0, O23.1, O23.2, O23.3, O23.4, O23.5, O23.9)*  ***O41.1*** *Infection of amniotic sac and membranes*  ***O85*** *Puerperal sepsis*  ***O86*** *Other puerperal infections (incl. O86.0, O86.1, O86.2, O86.3, O86.4, O86.8)*  ***O88.3*** *Obstetric pyaemic and septic embolism*  ***O91*** *Infections of breast associated with childbirth (incl. O91.0, O91.1)*  ***P36*** *Bacterial sepsis of newborn (incl. P36.0, P36.1, P36.2, P36.3, P36.4, P36.5, P36.8, P36.9)*  ***P39*** *Other infections specific to the perinatal period (incl. P39.0, P39.2, P39.3, P39.4, P39.8, P39.9)*  ***P78*** *Other perinatal digestives system disorders (P78.0, P78.1,)*  ***T814*** *Infection following a procedure, not elsewhere classified*  ***T845*** *Infection and inflammatory reaction due to internal joint prosthesis*  ***Certain Infectious and parasitic diseases***  ***A01*** *Typhoid and paratyphoid fevers (incl. A01.0, A01.1, A01.2, A01.3, A01.4)*  ***A02*** *Other salmonella infections (incl. A02.0, A02.1, A02.2, A02.8, A02.9)*  ***A03*** *Shigellosis (incl. A03.0, A03.1, A03.2, A03.3, A03.8, A03.9)*  ***A04*** *Other bacterial intestinal infections (incl. A04.0, A04.1, A04.2, A04.3, A04.4, A04.5, A04.6, A04.7, A04.8, A04.9)*  ***A06*** *Amoebiasis (incl. A06.0, A06.1, A06.2, A06.3, A06.4, A06.5, A06.6, A06.7, A06.8, A06.9)*  ***A15*** *Respiratory tuberculosis (incl. A15.0, A15.2, A15.3, A15.4, A15.5, A15.6, A15.7, A15.8, A15.9)*  ***A16*** *Respiratory tuberculosis, not confirmed bacteriologically or histologically (incl. A16.0, A16.1, A16.2, A16.3, A16.4, A16.5, A16.7, A16.8, A16.9)*  ***A17*** *Tuberculosis of nervous system (incl. A17.0, A17.1, A17.8, A17.9)*  ***A18*** *Tuberculosis of other organs (incl. A18.0, A18.1, A18.2, A18.3, A18.4, A18.5, A18.6, A18.7, A18.8)*  ***A19*** *Miliary tuberculosis (incl. A19.0, A19.1, A19.2, A19.8, A19.9)*  ***A27*** *Leptospirosis (incl. A27.0, A27.8, A27.9)*  ***A32*** *Listerosis (incl. A32.0, A32.1, A32.7, A32.8, A32.9)*  ***A37*** *Whopping cough (all subcategories)*  ***A38*** *Scarlet fever*  ***A39*** *Meningococcal infection (incl. A39.0, A39.1, A39.2, A39.4, A39.5, A39.8, A39.9)*  ***A42*** *Actinomycosis (all subcategories)*  ***A43*** *Nocardiosis (all subcategories)*  ***A44*** *Bartenollosis (all subcategories)*  ***A46*** *Erysipelas*  ***A48*** *Other Bacterial diseases, not elsewhere classified (incl. A48.0, A48.1, A48.2, A48.3, A48.4, A48.8)*  ***A49*** *Bacterial infection of unspecified site (incl. A49.0, A49.1, A49.2, A49.3, A49.8, A49.9)*  ***A51*** *Early syphilis (all subcategories)*  ***A54*** *Gonococcal infection (incl. A54.1, A54.2, A54.3, A54.4, A54.5, A54.6, A54.8, A54.9)*  ***A55*** *Chlamydial lymhogranuloma (venereum)*  ***A56*** *Other sexually transmitted chlamydial diseases (incl. A56.0, A56.1, A56.2, A56.3, A56.4, A56.8)*  ***A68*** *Relapsing fevers (all subcategories)*  ***A69.2*** *Lyme disease*  ***A70*** *Chlamydia psittaci infection*  ***A75*** *Typhus fever (all subcategories)*  ***A77*** *Spotted fever (all subcategories)*  ***A78*** *Q fever*  ***A79*** *Other rickettsioses (all subcategories)*  ***B59*** *Pneumocystosis*  ***Symptoms, signs and abnormal clinical and laboratory findings, not elsewhere classified***  ***R65*** *Systemic Inflammatory Response syndrome (incl. R65.0, R65.9)* |
| ***Septic shock/SIRs with organ dysfunction*** | ***R57.2*** *Septic shock*  ***R65.1*** *Systemic Inflammatory Response Syndrome of infectious origin with organ failure (severe sepsis)* |
